# Measuring Workflow Interruptions in Endoscopy: Development and Interrater Reliability of a Structured Observation Tool

**DOI:** 10.1055/a-2925-5508

**Published:** 2026-08-06

**Authors:** Christian Torres Reyes, Antonia Zörb, Elena Theiß, Nelina Dimitrova, Peter Ewald, Oscar Cahyadi, Alanna Ebigbo

**Affiliations:** 1 9142 Ruhr-Universität BochumBochumNRWGermany; 2 Department of Internal Medicine I (Gastroenterology) 91789 Katholisches Klinikum Bochum, Sankt Josef-HospitalBochumNRWGermany

**Keywords:** patient safety, human factors, workflow interruptions, workflow analysis, interrater reliability, team communication

## Abstract

**Background** Workflow interruptions are common in procedural medicine and may increase cognitive load, stress, and the risk of errors. While interruptions and flow disruptions have been studied in surgical settings, little is known about workflow interruptions in endoscopic environments. Objective evaluation of workflow interruptions requires a reliable measurement tool. This study aimed to develop a structured observation tool for recording workflow interruptions during endoscopic procedures and to evaluate its interrater reliability.

**Methods** A structured tally-based observation tool for recording workflow interruptions was developed in an iterative process, and interruptions were categorized into predefined domains. Three independent observers underwent structured training and calibration. A predefined subset of endoscopic procedures was observed simultaneously by two independent observers. Interrater reliability was assessed using intraclass correlation coefficients (ICCs) and Bland–Altman analysis.

**Results** A total of 35 procedures were scheduled for double observation, and 33 procedures were included in the final interrater reliability analysis. Interrater reliability for the total number of interruptions was excellent for both observer pairs (ICC 0.995 for A–B and 0.995 for A–C). Reliability across interruption domains ranged from good to excellent. Bland–Altman analysis demonstrated small mean differences with narrow limits of agreement and no relevant systematic bias.

**Conclusion** This methodologic study demonstrates that workflow interruptions during endoscopic procedures can be measured reliably using a structured tally-based observation tool. The instrument provides a standardized methodologic framework for future studies investigating workflow processes and workflow-focused interventions in endoscopy.

## Introduction

Endoscopy is a central component of modern gastroenterology and represents a complex procedural environment. Endoscopic procedures are performed by interdisciplinary teams under time pressure, often in patients with significant comorbidities and under sedation. In addition to the procedural task itself, parallel responsibilities, frequent communication, and external demands such as phone availability contribute to a highly dynamic workflow. These factors make endoscopy units particularly vulnerable to workflow interruptions and competing task demands. 1 2 3

In such complex environments, teamwork, communication, and workflow organization play a critical role in procedural quality and patient safety. 4 5 Workflow interruptions and flow disruptions have been associated with increased cognitive load, cause distraction, and negatively affect procedural performance. 6 7 8 9 10 11 12 While these aspects have been extensively studied in aviation and surgical settings, including the implementation of structured safety interventions such as crew resource management, checklists, and standardized communication protocols, 11 13 14 15 they have received comparatively little systematic attention in endoscopy. 16 17 18 19 20 21 Consequently, the effectiveness of workflow-focused interventions in endoscopy remains difficult to evaluate systematically.

Reliable measurement is a prerequisite for evaluating workflow interventions, benchmarking workflow quality, and assessing implementation strategies aimed at improving patient safety and procedural performance. 2 20 In surgical environments, validated observational frameworks have enabled systematic investigation of workflow interruptions and human-factor interventions. 8 11 In contrast, despite increasing interest in patient safety and human factors, endoscopy currently lacks a standardized and validated instrument for prospective quantification of workflow interruptions in routine practice, with only limited structured approaches. 19

Therefore, the aim of this methodological study was to develop and validate a structured observation tool for prospective assessment of workflow interruptions during endoscopic procedures, thereby providing a standardized methodologic framework for future intervention, implementation, and multicenter studies in endoscopy.

## Methods

### Study Setting

This methodologic study was conducted within the framework of the LIGHT study (Less Interruptions in Gastrointestinal Healthcare Teams), a prospective study investigating workflow interruptions in an endoscopy unit and evaluating a visual traffic light system to signal procedure status and critical phases.

The study was performed in the endoscopy unit of a tertiary care academic hospital in Germany. The unit performs diagnostic and interventional endoscopic procedures including esophagogastroduodenoscopy, colonoscopy, endoscopic ultrasound (EUS), and endoscopic retrograde cholangiopancreatography (ERCP). Interventional procedures including dilation, drainage, fine-needle biopsy, resections, and third-space endoscopies are performed daily. All procedures were performed by gastroenterologists and trainees with support from trained endoscopy nurses under endoscopist-directed sedation. No procedures were performed under general anesthesia.

The data for this analysis were collected during the baseline phase of the LIGHT study before implementation of the traffic light intervention. The general layout of the endoscopy rooms and typical observer positions are shown in **Supplemental Fig. 1** . Observers positioned themselves close to the wall with visibility of both the endoscopy team and the entrance door while minimizing interaction with the team. Observations were performed during routine clinical practice without modification of workflow.

### Development of the Observation Tool

The observation tool was developed pragmatically for real-time use in a live endoscopy environment. Its development was informed by previous observational studies on distractions and workflow disruptions in surgical and procedural settings and by established concepts of workflow interruptions in healthcare. 22 The aim was to create a simple tool that could be applied during routine procedures without technical equipment and with minimal subjective interpretation.

In an initial development phase, potentially relevant events were identified from the literature and from direct observation of routine endoscopic workflow. 7 8 10 Early versions included a more event-based documentation approach. However, this approach proved difficult to apply consistently because observers had to judge whether several observable occurrences represented one interruption episode or multiple separate events.

The tool was therefore simplified into a tally-based format that focused on predefined, directly observable events. Categories were iteratively refined during pilot use, and ambiguous categories were removed. Final category definitions were consolidated in a study-specific codebook.

Workflow interruptions were defined as observable events in the procedural environment that may disrupt ongoing tasks and shift attention away from the primary task. 23 The tool therefore captures disruptive events in the procedural environment rather than only interruptions with confirmed task cessation.

The final version of the tool was tested by simultaneous observation of 10 endoscopic procedures by different observers. After each procedure, discrepancies between observers were discussed and definitions standardized. The observation form was implemented as a structured data-collection instrument in REDCap. The final observation sheet and codebook are provided in the Supplementary Material to facilitate reproducibility.

Initially, categorization of team members’ level of experience was planned. Following recommendations from the ethics committee, this variable was removed due to concerns regarding potential re-identification in a single-center team setting.

### Observer Training and Calibration

After finalization of the codebook, observer training was performed. Training consisted of theoretical instruction, explanation of each interruption category using examples, including discussion of potentially ambiguous scenarios, and calibration through simultaneous observation of 10 endoscopic procedures. Discrepancies between observers were discussed and definitions standardized to ensure consistent interpretation of interruption categories.

Observations were performed by one primary observer and two additional trained observers with different professional backgrounds (medical student, study nurse, and scientific assistant with medical training). All observers underwent the same training and calibration process prior to data collection.

### Study Design and Outcome

Data were collected using a tally-based real-time observation and documented on standardized paper forms, an established approach for analyzing workflow and interruptions in clinical environments. 6 During the four-week baseline observation period of the LIGHT study, a predefined subset of procedures was simultaneously observed by a secondary observer to assess interrater reliability.

No formal sample size calculation was performed for the interrater reliability analysis. The number of double-observed procedures was determined pragmatically based on feasibility during the baseline phase and was considered sufficient to assess agreement for total interruption domains across two observer pairs.

Observers recorded interruptions independently without communication during procedures. During simultaneous observations, observers were positioned in different areas of the procedure room to ensure independent observation while maintaining visual access to the team and environmental events such as room entry and phone activity.

The primary outcome was the agreement in the total number of interruptions per procedure between observers. Secondary outcomes included agreement in the number of interruptions within each interruption category.

Procedures with protocol violations (e.g., communication between observers during observation) were excluded from the interrater reliability analysis.

The reporting of this methodologic study was guided by the Guidelines for Reporting Reliability and Agreement Studies (GRRAS), where applicable.

### Definition of Interruption Domains

Individual interruption events were grouped into predefined domains to facilitate analysis and improve interpretability ( **Supplemental Table 1** ), reflecting commonly described sources of workflow interruptions in healthcare. 23 Environmental interruptions included door openings and phone ringing. External communication included phone use by team members and communication with persons outside the team. Internal team communication is presented as an overall category and additionally described by procedure-related and non-procedure-related communication, which were recorded as separate, nonmutually exclusive communication episodes. To improve consistency, examples of both categories were included in the observer codebook and discussed during calibration sessions. Procedure-related communication referred to verbal exchanges directly related to the ongoing procedure, patient management, or procedural decision-making. Non-procedure-related communication referred to conversations unrelated to the current procedure, such as organizational issues unrelated to the ongoing procedure, scheduling of subsequent procedures, or private topics. In cases where communication contained both procedural and nonprocedural elements, observers were instructed to classify the dominant focus of the interaction.

Movement-related interruptions included nurse leaving the room, physician leaving the room, and team member change.

The total number of interruptions per procedure was calculated as the sum of all recorded interruption events across all categories.

### Statistical Analysis

Statistical analysis followed a predefined statistical analysis plan. Analyses were performed using R (R Foundation for Statistical Computing, Vienna, Austria, Version 4.5.3).

Interrater reliability was assessed for the total number of interruptions per procedure as well as for predefined interruption categories. Agreement between observers was evaluated using the intraclass correlation coefficient (ICC) based on a two-way random-effects model with absolute agreement and single measurements. 24 25 ICC values were reported with 95% confidence intervals.

Agreement was interpreted according to commonly used thresholds: <0.50 poor, 0.50–0.75 moderate, 0.75–0.90 good, and >0.90 excellent reliability.

In addition, agreement between observers was assessed using Bland–Altman analysis. 26 Mean differences and limits of agreement (mean difference ± 1.96 standard deviations) were calculated and visualized using Bland–Altman plots.

Continuous variables were summarized using mean ± standard deviation or median and range as appropriate.

As this was a methodologic study focusing on agreement rather than hypothesis testing, interpretation was primarily based on ICC values, confidence intervals, and Bland–Altman analysis. Given that interruption counts represent continuous variables, ICC was considered more appropriate than Cohen’s kappa.

## Results

### Double-observed Procedures

A total of 35 procedures were scheduled for double observation. Two procedures were excluded due to protocol violations (observer communication), resulting in 33 procedures included in the interrater reliability analysis. Of these, 12 procedures were assessed by observer pair A–B and 21 by observer pair A–C. Characteristics of the double-observed procedures are summarized in Table 1 .

**Table 1:** Characteristics of double-observed endoscopic procedures included in the interrater reliability analysis.

Variable	Overall ( *n* = 33)	A vs. B ( *n* = 12)	A vs. C ( *n* = 21)
Procedure duration, mean ± SD, min	16.8 ± 14.8	15.6 ± 13.3	17.4 ± 15.9
Procedure duration, median (range), min	10 (5–53)	11 (5–53)	9 (5–55)
Total interruptions, mean ± SD	37 ± 28.6	25.7 ± 16.9	43.4 ± 32.1
Total interruptions, median (range)	25 (8–109)	21 (8–68)	33 (9–109)

Values are presented as mean ± standard deviation or median (range).

The distribution of interruption categories is shown in Table 3 . Communication-related interruptions accounted for the largest proportion, whereas movement-related interruptions were less frequent.

### Interrater Reliability

Interrater reliability for the total number of interruptions was excellent for both observer pairs (ICC 0.995 for A–B and 0.995 for A–C). Reliability across interruption domains ranged from good to excellent. The lowest agreement was observed for procedure-related communication in observer pair A–B (ICC 0.912) and the highest agreement was observed for environmental interruptions in the same pair (ICC 0.998). Detailed ICC values are presented in Table 2 .

**Table 2:** Interrater reliability for interruption categories assessed by intraclass correlation coefficients (ICC).

Category	A vs. B ICC (95% CI)	A vs. C ICC (95% CI)
Total interruptions	0.995 (0.982–0.998)	0.995 (0.988–0.998)
Environmental interruptions	0.998 (0.993–0.999)	0.966 (0.918–0.986)
Internal team communication	0.972 (0.906–0.992)	0.994 (0.985–0.998)
Procedure-related communication	0.912 (0.734–0.973)	0.987 (0.969–0.995)
Non-procedure-related communication	0.988 (0.960–0.997)	0.990 (0.976–0.996)
External communication	0.953 (0.824–0.987)	0.960 (0.876–0.985)
Movement	0.983 (0.945–0.995)	0.936 (0.851–0.973)

ICC = intraclass correlation coefficient; CI = confidence interval.

ICCs were calculated using a two-way random-effects model for absolute agreement (single measurements).

**Table 3:** Number of interruptions per category across all double-observed endoscopic procedures based on the primary observer.

Category	Mean ± SD	Median (range)
Environmental interruptions	4.8 **±** 4.3	4 (0–16)
Internal team communication	24.4 **±** 20.4	17 (4–82)
Procedure-related communication	14 **±** 11.2	10 (3–46)
Non-procedure-related communication	23 **±** 18.6	16 (3–71)
External communication	6.4 **±** 6.5	4 (0–28)
Movement	1.4 ± 1.5	1 (0–5)

SD = standard deviation.

Values are presented as mean **±** standard deviation or median (range).

### Agreement Analysis

**Fig. 1:**
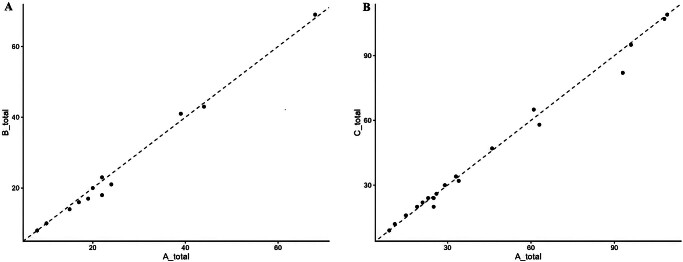
Scatterplots showing agreement in total interruption counts between observers. ( **A** ) Observer pair A–B. ( **B** ) Observer pair A–C. The dashed line represents the line of identity.

**Fig. 2:**
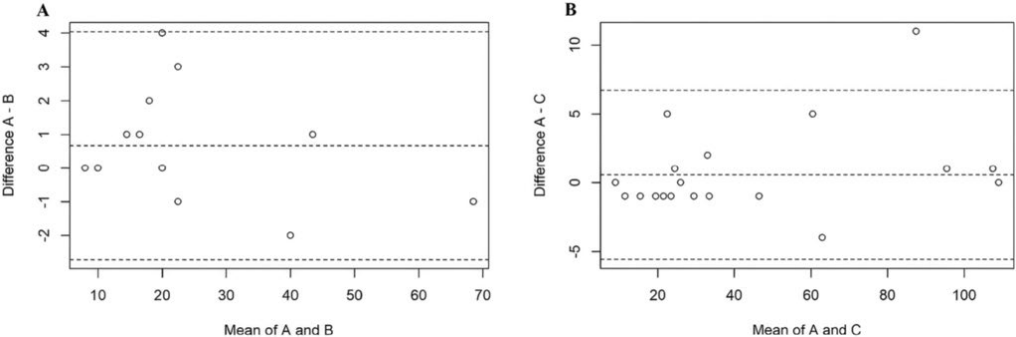
Bland–Altman plots showing agreement in total interruption counts between observers. ( **A** ) Observer pair A–B. ( **B** ) Observer pair A–C. The central dashed line represents the mean difference, and the dashed lines indicate the limits of agreement (mean difference ±1.96 standard deviations).

Agreement between observers is illustrated in scatterplots ( Fig. 1 ). Bland–Altman analysis demonstrated small mean differences with narrow limits of agreement. For observer pair A–B, the mean difference was 0.67 interruptions (limits of agreement −2.71 to 4.04). For observer pair A–C, the mean difference was 0.57 interruptions (limits of agreement −5.58 to 6.73) ( Fig. 2 ).

The median absolute difference between observers was 1 interruption for both observer pairs. Absolute differences ranged from 0 to 4 interruptions for A–B and from 0 to 11 interruptions for A–C. Exact agreement was observed in 25% of procedures for A–B and 14% for A–C.

## Discussion

In endoscopy, scientific focus has traditionally been placed on technical and procedural outcomes, whereas workflow processes and human factors have received comparatively little attention. However, workflow interruptions may increase cognitive load, stress, and the risk of errors in procedural environments, 6 12 27 highlighting their potential relevance for procedural quality and patient safety.

This methodologic study was designed to address a fundamental methodologic gap in endoscopy research: the lack of a standardized instrument for prospective assessment of workflow interruptions. Reliable measurement represents a prerequisite for evaluating workflow interventions, implementation strategies, and human-factors concepts in endoscopy. The present study demonstrates that a structured, tally-based observation tool can reliably quantify workflow interruptions during routine endoscopic procedures.

The high level of agreement observed in this study is likely related to the low-inference design of the observation tool, which focuses on directly observable events rather than subjective interpretation. The use of predefined categories and a tally-based approach reduced ambiguity and facilitated consistent event recording. In contrast, more complex event-based documentation approaches may require interpretation of interruption episodes and are therefore more prone to variability between observers.

High-quality endoscopy does not only depend on intraluminal quality indicators such as adenoma detection rate, withdrawal time, or completeness of examination, 2 but also on external workflow factors including team communication, interruptions, and procedural organization. 20 28 The presented observation tool provides a structured method to quantify these aspects and may complement traditional quality indicators in endoscopy.

Beyond reliability, the main value of the observation tool lies in its ability to provide a standardized outcome measure for workflow-focused research. Similar to established quality indicators in endoscopy, structured measurement is necessary before workflow interventions can be rigorously evaluated. The present tool was specifically developed to support future studies investigating the relationship between workflow interruptions, procedural performance, team processes, and patient-related outcomes.

Procedure duration was similar between observer pairs, whereas the number of recorded interruptions differed. This likely reflects variability in team composition, communication behavior, procedure type, and exposure to external interruptions under real-life conditions rather than observer-related effects. 27 29 Importantly, interrater reliability remained consistently high despite these differences.

Exact agreement was not observed in all procedures, which is expected in real-time observation of dynamic clinical environments. However, the median absolute difference between observers was small and no relevant systematic bias was detected, supporting the robustness of the observation tool.

The simplicity of the tool represents an important advantage. It can be applied in routine clinical practice without technical equipment and can be used by observers with different professional backgrounds after structured training and calibration. This enables low-threshold implementation for both research and quality improvement purposes.

A further advantage of the observation tool is its pragmatic design. In contrast to approaches relying on video analysis, specialized software, or extensive postprocedural coding, the present instrument can be implemented using direct real-life observation with minimal technical requirements. This lowers barriers to adoption and facilitates application across different endoscopy settings. Beyond research applications, the tool may support local quality-assurance initiatives, benchmarking of workflow characteristics between units, and systematic evaluation of workflow-focused interventions. Its low-resource design may be particularly advantageous for prospective implementation and multicenter studies, where feasibility and reproducibility are essential.

This study has several strengths. It was conducted prospectively in a real-life endoscopy setting and included different procedure types and room environments. The structured development process, observer training, and calibration resulted in a feasible observation tool with excellent interrater reliability that can be implemented without technical equipment. These findings strengthen the methodologic basis of the LIGHT study.

Several limitations should be considered. The study was conducted in a single tertiary endoscopy unit, which may limit generalizability. All procedures were performed under procedural sedation, and interruption patterns may differ in settings using general anesthesia. The number of double-observed procedures was limited, although sufficient for reliability assessment. Observer positioning and the lack of video recording may have influenced the number of recorded interruptions; however, video-based assessment was not feasible due to ethical and data-protection considerations. A Hawthorne effect cannot be excluded, although efforts were made to minimize behavioral changes during observation. Furthermore, all observers were aware of the study objective. Although structured training was necessary to ensure consistent event classification, observer-related behavioral effects cannot be fully excluded. Information on team experience was intentionally not collected following ethics committee recommendations to avoid potential re-identification of staff members in a single center setting.

Although interruption frequencies may differ between institutions, the low-inference design and reliance on directly observable events may facilitate transferability across different endoscopy settings. Furthermore, the reliance on directly observable events rather than institution-specific workflow metrics may facilitate adaptation to different staffing models and procedural settings.

Future interventional and multicenter studies may use this instrument to evaluate targeted workflow interventions, human factor training concepts, and implementation strategies designed to reduce unnecessary interruptions and optimize procedural workflows, as demonstrated in other clinical settings. 27 Establishing a reliable measurement framework represents an essential prerequisite before the effectiveness of workflow-focused interventions can be rigorously evaluated.

Beyond research applications, the tool may also be used for workflow assessment and quality assurance in endoscopy units, consistent with system-based approaches to quality and safety improvement. 30 Intermittent observations may be sufficient to identify common sources of interruptions and evaluate workflow improvement measures. As the tool does not require technical equipment, implementation is feasible with relatively low resources, although real-time observation requires dedicated personnel. Future developments such as digital data-collection tools or automated detection methods may further facilitate workflow analysis.

The validated observation tool provides the methodologic foundation for subsequent analyses within the LIGHT study and for a broader research program investigating workflow optimization, implementation strategies, and human-factor interventions in endoscopy.

## Conclusions

While technical quality indicators in endoscopy such as adenoma detection rate, withdrawal time, or complication rates are well established, workflow and environmental factors have received less attention. The present observation tool provides a structured method to quantify workflow interruptions and procedural environmental factors and may complement traditional quality indicators in endoscopy.

The structured tally-based observation tool represents a feasible and reliable method for quantifying workflow interruptions during routine endoscopic procedures. It demonstrated excellent interrater reliability and enables objective and reproducible measurement of workflow interruptions. The tool provides a standardized and reproducible method for assessing workflow interruptions and may facilitate future clinical studies and quality-improvement initiatives aimed at optimizing endoscopic workflows.

## Supplementary Material

Ergänzendes Material Supplementary Material
