# Supplementary material for: Measuring Workflow Interruptions in Endoscopy: Development and Interrater Reliability of a Structured Observation Tool
Source: Endosc Int Open. 2026 Aug 6;14:a29255508. doi: 10.1055/a-2925-5508 (PMC13463359; doi:10.1055/a-2925-5508)
Supplement: Supplementary file 1 [file 10-1055-a-2925-5508_29262874.pdf]

Thieme

Supplementary Material: Measuring workflow interruptions in endoscopy: development and interrater reliability of a structured observation tool

List of supplementary material:

1. Supplemental Table 1. Grouping of observed interruption events into interruption domains
2. Observation tool
3. Codebook

Endosc Int Open | DOI 10.1055/a-2925-5508 | © The Author(s).

Supplemental Table 1. Grouping of observed interruption events into interruption domains

Interruption domain	Observed event
Environmental interruptions	Door opening
	Phone ringing
External communication	Nurse answering phone
	Physician answering phone
	Communication with persons outside the team
Internal team communication	Procedure-related communication within the team
	Non-procedure-related communication within the team
Movement	Nurse leaving the room
	Physician leaving the room
	Team member change

Thieme

Observation Tool

Record ID	______________________________
primary observer	○ Yes<br>○ No
Observer	○ A<br>○ B<br>○ C
Date of observation	______________________________
Endoscopy room	○ EGD<br>○ Colonoscopy<br>○ Fluoroscopy<br>○ EUS
Procedure type	☐ EGD<br>☐ Colo<br>☐ ERCP<br>☐ EUS<br>☐ Bronchoscopy<br>☐ Other
Intervention	○ Yes<br>○ No
Intervention - type	
Start time	
End time	
Duration of procedure	
door opening	
phone ringing	
Nurse answering phone	

Endosc Int Open | DOI 10.1055/a-2925-5508 | © The Author(s).

Physician answering phone
Communication with persons outside the team
Procedure-related communication within the team
Non-procedure-related communication within the team
Physician leaving the room
Nurse leaving the room
Team member change

Endosc Int Open | DOI 10.1055/a-2925-5508 | © The Author(s).

Codebook

The observation tool was designed as a low-inference tool focusing on directly observable events rather than subjective interpretation.

1. General Observation Rules

Observation period

- The observation period begins with the first administration of sedatives.
- If no sedatives are used, the observation period begins with the first use of an endoscopic instrument.
- The observation period ends as soon as the last endoscopic instrument leaves the patient's body for the last time.

General definition of interruptions

Interruptions are defined as observable events in the procedural environment that have the potential to disrupt workflow, including events that trigger an observable behavioral response as well as clearly perceivable events that could potentially distract the team even without a visible reaction.

Counting rules

- Each occurrence of an event is counted separately.
- If multiple events occur simultaneously, each event is counted individually.
- Continuous conversations are counted as one conversation episode.
- No weighting of interruptions was applied.

Endosc Int Open | DOI 10.1055/a-2925-5508 | © The Author(s).

2. Interruption Categories and Definitions

Category	Definition	Examples	Counting Rule
Door opening	Opening of the endoscopy room door during a procedure	Staff opens the door to leave or enter the room, the door is briefly opened	Count each door opening separately
Unanswered phone ringing	Phone rings in the room but is not answered	Ward calls but nobody answers	One continuous phone ringing is counted as a single event
Phone use by nurse	Phone call is answered or initiated by a nurse, a trainee or a student	Nurse answers ward phone call, calls ward to order a patient, takes over a phone call from another person	Count when the phone conversation begins
Phone use by physician	Phone call is answered or initiated by physician	Physician receives call, calls another person or takes over a phone call from another person	Count when the phone conversation begins
Communication with persons outside the team	Any conversation with or among persons not directly involved in the procedure	Conversation with or among ward staff, technicians, external physicians in the room	Count each communication episode
Procedure-related team communication	Communication within the team directly related to the procedure	Instrument requests, discussion of findings, teaching, sedation-related communication, workflow coordination directly related to the ongoing procedure	Count each communication episode

Endosc Int Open | DOI 10.1055/a-2925-5508 | © The Author(s).

Category	Definition	Examples	Counting Rule
Non-procedure-related team communication	Communication within the team not related to the procedure	Small talk, private conversation, discussions unrelated to patient care or procedural workflow including immediate procedural workflow (e.g. preparation of the next patient or equipment required for the next scheduled procedure).	Count each communication episode
Nurse leaving room	Nurse, trainee or student leaves the procedure room	Nurse leaves to get equipment	Count each exit
Physician leaving room	Physician leaves the procedure room	Physician leaves for phone call	Count each exit
Team member change	Replacement or addition of team member	New nurse replaces another, another physician is added to the team	Count each change
Other interruption	Any interruption not covered by predefined categories	Equipment failure, alarm sound	Each event should be described and counted separately. They are not to be included in predefined interruption domains.

If a communication episode contained both procedure-related and non-procedure-related elements, observers were instructed to classify the dominant focus of the interaction. Such situations were discussed during observer training and calibration sessions.

Endosc Int Open | DOI 10.1055/a-2925-5508 | © The Author(s).

3. Team Definition

The procedural team is defined as the endoscopist, assisting nurses and trainees or students present in the room at the start of the procedure. Persons entering the room temporarily are considered external to the team regardless of their professional role.

4. Special Rules

Simultaneous events

If a door opening and a conversation occur simultaneously, both events are counted separately.

The opening of the door is counted separately to other events. For example, if an assisting nurse opens the door to leave the room, both events are counted.

Conversations

A continuous conversation is counted as one communication episode. If the conversation stops and restarts later, it is counted as a new episode.

Door left open

The door being left open is not counted continuously; only the opening event itself is counted. If a person enters the room, leaves the door open and later exits through the open door, the opening of the door is only counted once, when it is actually opened.

Endosc Int Open | DOI 10.1055/a-2925-5508 | © The Author(s).

Supplemental Figure 1

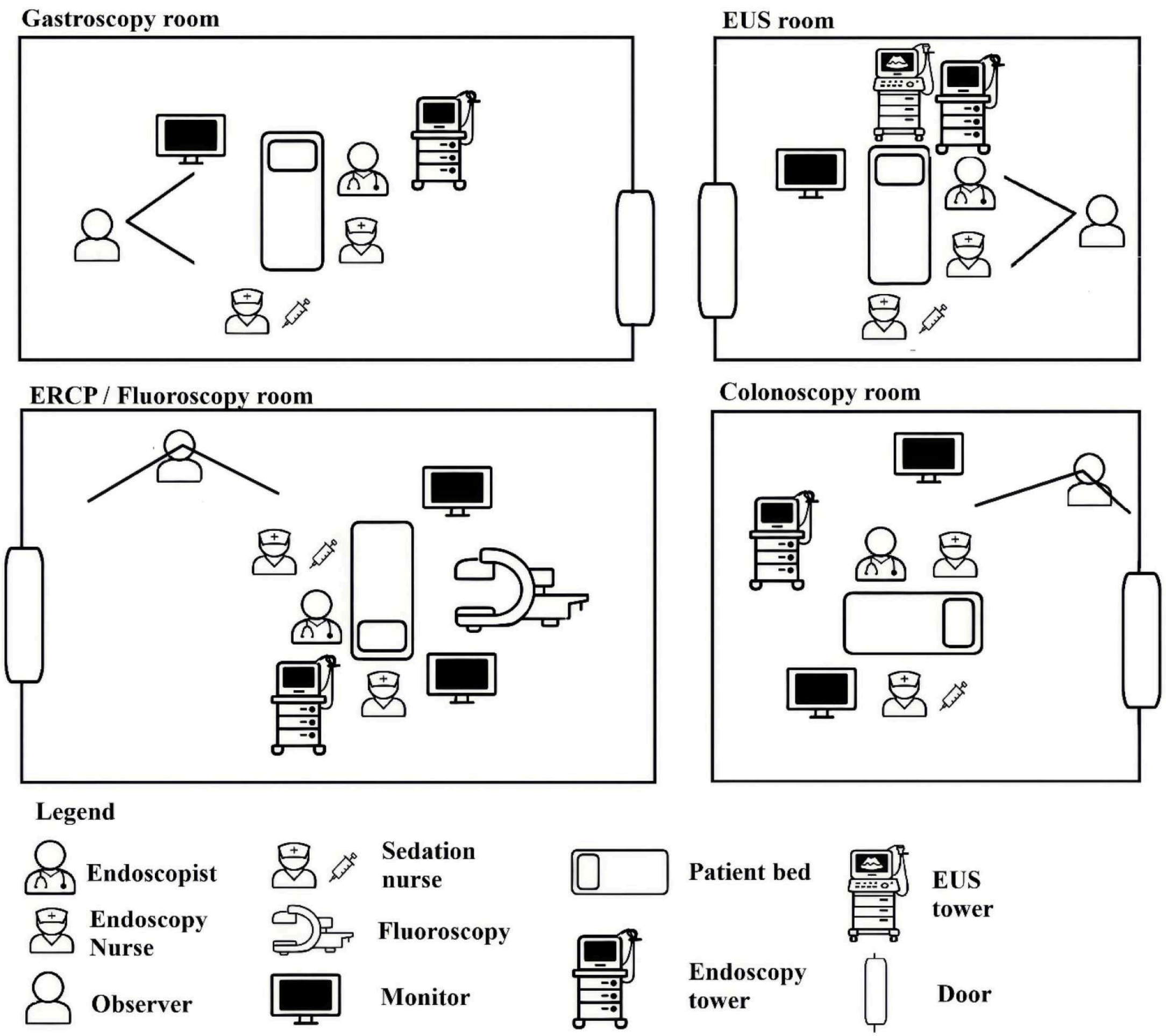

Endosc Int Open | DOI 10.1055/a-2925-5508 | © The Author(s).
